# Bilateral lung nodules resection by image-guided video-assisted thoracoscopic surgery: a case series

**DOI:** 10.1186/s13019-020-01253-5

**Published:** 2020-07-29

**Authors:** Chu-Chun Liang, Chi-Hao Liao, Ya-Fu Cheng, Wei-Heng Hung, Heng-Chung Chen, Chang-Lun Huang, Bing-Yen Wang

**Affiliations:** 1 Division of Thoracic Surgery, Department of Surgery, Changhua Christian Hospital, No. 135 Nanxiao St., Changhua City, Changhua County 500 Taiwan; 2 School of Medicine, College of Medicine, Kaohsiung Medical University, Kaohsiung, Taiwan; 3 Institute of Genomics and Bioinformatics, National Chung Hsing University, Taichung, Taiwan; 4 Program in Translational Medicine, National Chung Hsing University, Taichung, Taiwan; 5 School of Medicine, Chung Shan Medical University, Taichung, Taiwan; 6 Center for General Education, Ming Dao University, Changhua, Taiwan

**Keywords:** Image-guided video-assisted thoracoscopic surgery, Hybrid operating room, Small pulmonary nodules

## Abstract

**Background:**

We demonstrated the safety and feasibility of image-guided video-assisted thoracoscopic surgery (iVATS) of bilateral lung lesions in a hybrid operating room.

**Methods:**

This study was a retrospective analysis of a case series. A total of 7 patients with 15 small lung nodules underwent bilateral iVATS between July 2018 and May 2019. All procedures were completed within a single anesthesia procedure and performed in a hybrid operating room that had a cone-beam computed tomography (CT) apparatus equipped with a laser navigation system. The lesion characteristics, operation methods, and peri-operative clinical outcomes were summarized.

**Results:**

A total of 7 patients with 15 resected lung nodules were analyzed. The most common pathological result of our bilateral iVATS was metastasis. The median length of hospital stay was 5 days (range from 3 to 10 days). The median right chest tube duration was 2 days (range from 1 to 8 days), and the median left chest tube duration was 3 days (range from 2 to 5 days). Only one patient had a complication during his hospitalization period. There was no surgery-related mortality observed.

**Conclusions:**

For bilateral pulmonary nodules, the iVATS procedure seems to be a feasible and cost-effective approach.

## Introduction

Over the past decades, video-assisted thoracoscopic surgery (VATS) resection for small lung nodules has been adopted more and more widely because of its optimized perioperative outcome and its acceleration of postoperative recovery [1, 2]. However, small lung nodules, especially ground glass opacities (GGOs), are difficult to palpate and obtain during the surgery. Accurately localizing small lesions before the operation is thus an important issue [1–10]. Several techniques have been developed for localizing peripheral small lung lesions; the list of techniques includes percutaneous image-guided injection of a dye (methylene blue, patent blue vital dye, barium sulphate, etc.) and image-guided placement of microcoils or hook wires [1–3, 6–8]. In the traditional way, patients were sent to an imaging room (usually guided by multidetector CT) for the lesion localization and then transferred to an operating room for the surgery. Nonetheless, concerns had been issued concerning the dislodgement of markers, the dissemination of dyes to lung parenchyma during patient transferal and the procedure gap between localization and surgery [2, 3, 8].

Several studies about image-guided video assisted thoracoscopic surgery (iVATS) have focused on the application of hybrid operating rooms, which provide multimodality and real-time imaging guidance to improve the localization problems and surgical procedures [1–6]. Those studies developed iVATS workflows to combine lesion localization and resection in a single surgical session. Intraoperative nodule localization allows one to complete localization and surgery without an anesthesia gap. Therefore, patient transferal and repositioning, marker dislodgement, and the potential risks of bleeding and pneumothorax are avoided [1–3, 6]. The overall perioperative outcomes are comparable to traditional CT-room-guided surgery and makes iVATS an attractive alternative [2–6]. However, the application of iVATS for bilateral pulmonary nodule localization and resection has not been explored. Patients who underwent bilateral lung nodule resection suffered from longer surgical duration and hospital stays and higher risks of anesthesia-related complications, pneumothorax and hemothorax, and moreover, there were more concerns about loss of lung functions. The advantages of iVATS in a hybrid room especially provide extra benefits for patients needing bilateral lung nodule resection.

In this case series, we described the feasibility and safety of bilateral small lung nodule resection via iVATS in a hybrid room. To our best knowledge, this article was also the first study focusing on a bilateral application of iVATS.

## Material and methods

### Patients

We did a thorough chart review retrospectively from July 2018 to May 2019. There were 109 patients receiving iVATS surgery for their lung nodules, and 7 of them had bilateral iVATS. The inclusion criteria for our study were (1) presence of a GGO > 5 mm in size with or without solid components on follow-up CT images, (2) enlarged nodule size during follow-up periods, (3) distance of the lesion to the pleura less than 3 cm, and (4) suspected metastatic lung lesion no matter patients who receive previous thoracic surgical interventions or not. Patients were excluded from review if they were (1) under 18 years old, (2) received unilateral iVATS surgery only, or (3) refused to sign the consent of research protocol. The Institutional Review Board of the Changhua Christian Hospital approved the study protocol (IRB-191201).

### iVATS surgical technique

**Fig. 1:**
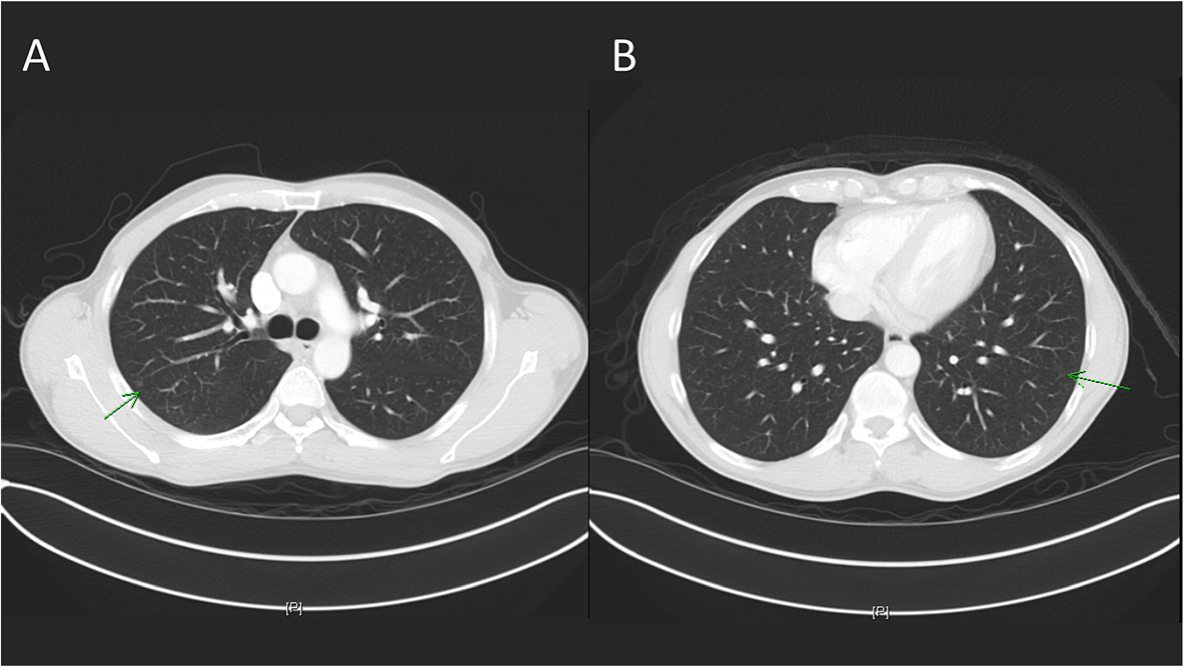
**a** The lung nodule of #2 patient at right upper lobe. **b** The lung nodule of #2 patient at left lower lobe

**Fig. 2:**
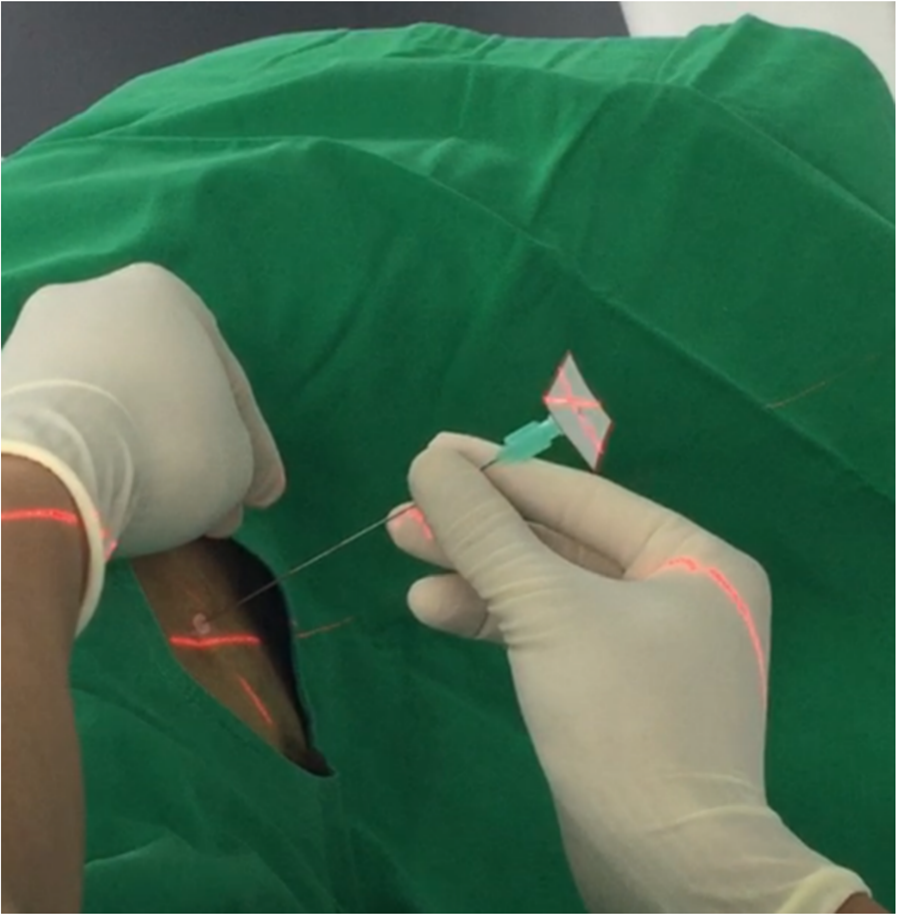
The localization of lung nodule at hybrid operative room under Artis pheno

**Fig. 3:**
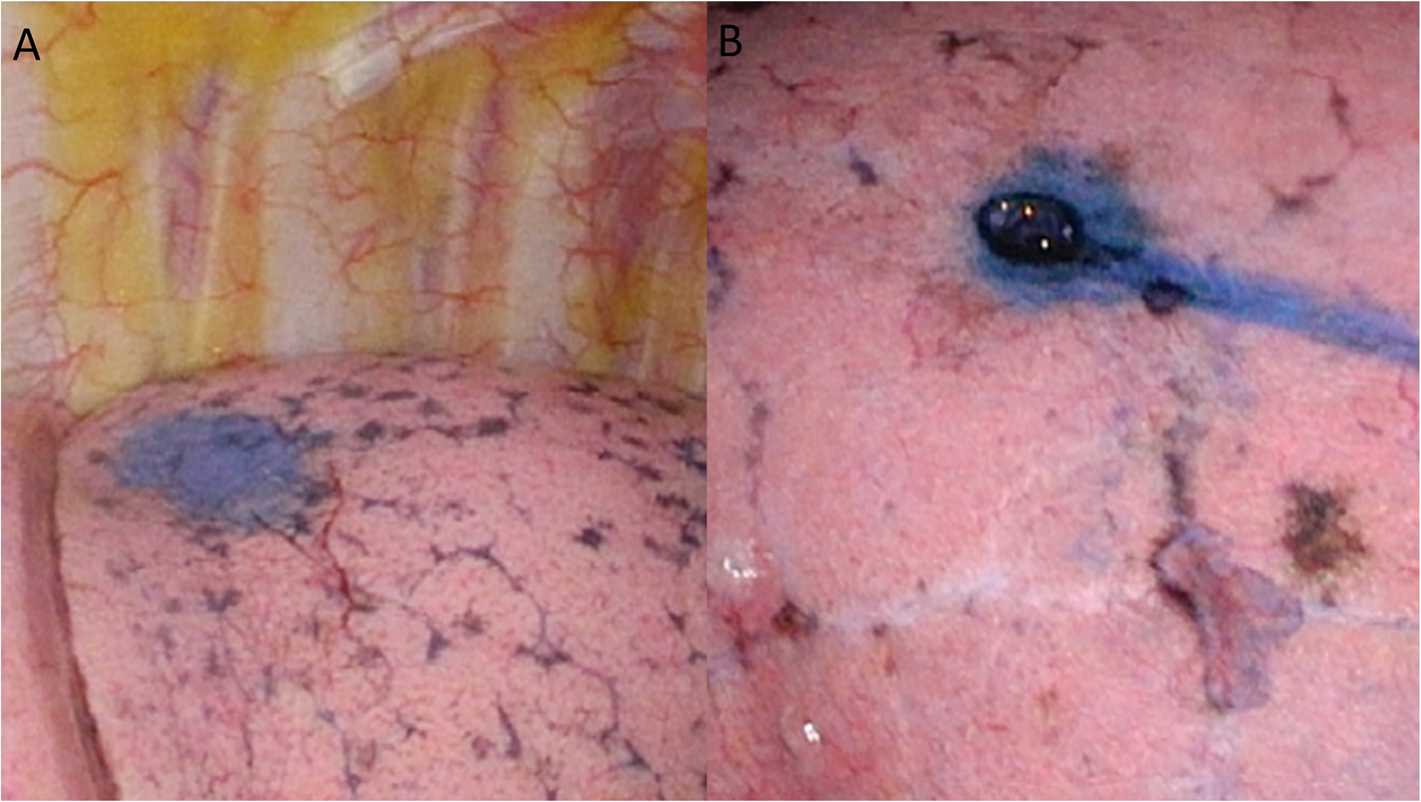
**a** The lung nodule of #2 patient under VATS at right upper lobe. The lung nodule of #2 patient under VATS at left lower lobe

All patients were admitted 1 day before surgery for preoperative preparation and management. At the initiation of the whole procedure, general anesthesia with double lumen intubation technique was introduced and our patients were then positioned in a lateral decubitus position, depending on the location of the lesion, for the dye localization and iVATS surgery. When deciding on which laterality to start, we chose the easier side or expected smaller resection side first in order to maintain maximal pulmonary volume and function of oxygenation during the other side’s procedure. We performed the surgery with a robotic C-arm cone beam CT (Artis pheno; Siemens Healthcare GmbH, Forchheim, Germany). The localization procedure was performed by one experienced chest surgeon (BY Wang). All patients were held a breath by suspension the ventilator at end-inspiratory phase. We then clamped the double lumen endotracheal tube by Kelly temporary. The cone beam CT scan was done and the shortest route for needle insertion to localize the lesion was then planned (Fig. 1). The route was projected with a laser beam on the skin of the patient under the syngo Needle Guidance of a syngo X-Workplace with a three-dimensional view. After measuring the depth, we introduced an 18-gauge coaxial needle into the patient’s chest under the guidance of the cone beam CT during end inspiration breath-hold phase (Fig. 2). A diluted methylene blue dye (0.2 ml) plus normal saline (0.3 ml) were subsequently injected into the lung lesion through the needle. A post-procedural cone-beam CT scan was obtained to confirm an appropriate needle location and make sure no further complication (pneumothorax or hemothorax) arose. After finishing the localization, the patient was positioned in the same position on the table and completely sterilized and then underwent thoracoscopic wedge resection (Fig. 3). All the resections were performed with single-incision VATS. We performed wedge resections for the peripheral nodules. Otherwise, segmentectomy was done for the central nodules to ensure safe margins. We performed a frozen section examination for every nodule after resection for the confirmation of the pathology. The frozen section can provide us the information of further operative choice. If primary lung cancer was approved by the frozen section, additional mediastinal lymph nodes dissection would be completed. We also had to decide whether further resection should be done to make sure the safe margin over 2 cm. On the other hand, no further resection or mediastinal lymph nodes dissection would be done if the metastatic cancer or benign nodule was diagnosed.

For the lesion of the contralateral lung, the same scanning and localization procedures were processed again and then the lesion was resected. The total operative time was measured from the initiation of image-guided localization to the end of the whole surgery (after final suturing for epidermis closure). All procedures were completed in a single general anesthesia event in one hybrid operative room.

### Data collection and statistical analysis

Due to the payment protocol of our national health insurance, we were not able to arrange preoperative positron emission tomography (PET) unless the diagnosis of cancer was confirmed. We arranged first postoperative chest CT follow-up one month after hospital discharge, followed by every 3 month till one year after surgery.

The study variables summarized in our series include patients’ clinical demographic data (age, gender), lesion characteristics (laterality, size, depth of nodule to pleura, pathological results), operation methods (wedge resection, segmentectomy, or lobectomy), and peri-operative clinical outcomes (length of hospital stay, chest tube duration, postoperative complication). They were all extracted from clinical documentation.

## Results

### Patient demographics and Clinicopathologic characteristics

All the background information and perioperative details of our patients were listed in Table 1. A total of 7 patients with 15 resected lung nodules were enrolled and analyzed in our series; 3 of them were females and 4 of them were males. The median age was 60 years old (ranged from 47 to 68 years old). Only one patient had two lesions over his right lung; others had one lesion over each side of their lungs. The median operative time for bilateral nodules was 245 min (ranged from 165 to 345 min). The median nodule size of the right lung was 8.5 mm (range from 5 to 11 mm), and the median nodule size of the left lung was 11 mm (range from 6 to 15 mm). The median distance from the right pulmonary nodule to the pleural surface was 10.5 mm (range from 5 to 14 mm), and the median distance from the left pulmonary nodule to the pleural surface was 17 mm (range from 7 to 26 mm). Among the 15 nodule resections, 3 of them were segmentectomies, and the other 12 were wedge resections.

**Table 1:** Clinical demographic data and management

Case	Age	Gender	Tumor location	OP time (min)	Nodule size (mm)	Distance to the pleura (mm)	*Pre-OP FEV1*
1	60	Female	RLL + LLL	296	9 / 7	13 / 9	1.65
2	47	Male	RUL + LLL	231	6 / 7	5 / 7	2.02
3	64	Female	RUL + LLL	345	5 / 12	14 / 22	1.58
4	60		RLL + LLL	265	8 / 11	11 / 24	1.82
5	63	Male	RUL + RLL + LLL	165	7 / 9 / 6	6 / 13 / 10	1.88
6	57	Male	RML + LUL	245	11 / 15	10 / 26	1.74
7	68	Male Female	RLL + LUL	197	9 / 12	10 / 17	1.48

*OP* operative, *RUL* right upper lobe, *RML* right middle lobe, *RLL* right lower lobe, *LUL* left upper lobe, *LLL* left lower lobe

Table 2 presented the pathological results and clinical outcomes of our patients. The majority of the lesions resected in our study were metastatic lesions (e.g., 8 of 15 lesions had hepatocellular carcinoma); 5 were primary lung cancers (3 were adenocarcinomas, one was adenocarcinoma in situ, and the other one was squamous cell carcinoma). Only two lesions were benign. All lesions in our study were partially solid or mixed ground glass, and their average size was 8.93 mm. The average length of hospital stay was 5.43 days (ranged from 3 to 10 days). On average the right chest tube duration was 3 days, and the average left chest tube duration was 3.43 days. Furthermore, only one of the 7 patients had a complication (right hydrothorax) during his hospitalization period. Only one of the 7 patients had a complication (right hydrothorax) during her hospitalization period. Due to prolonged chest tube amount around 500 to 1000 ml, we did not remove the right chest tube which placed during operation till postoperative day 8. At postoperative day 8 the amount of pleural effusion was less than 300 ml. We removed her chest tube after confirming his acceptable lung expansion by chest x-ray and he was discharged smoothly. No further invasive intervention was done. The intraoperative hemodynamics and oxygen saturation were stable. No complication such as desaturation, hypotension was noted of these 7 patients. We routinely followed up radiographic both 1 day before chest tube removal and 1 day before hospital discharge.

**Table 2:** Outcomes of bilateral localization at hybrid room

Case	OP method	Pathology	LoS (day)	Length of chest tube removal (day)	Complication
1	RLL: wedge, LLL: wedge	RLL,LLL: SCC, metastasis	10	R: 8, L: 3	Right hydrothorax
2	RUL: wedge, LLL: wedge	RUL: AIS, LLL: inflammation	3	R: 1, L: 2	None
3	RUL: wedge, LLL: segmentectomy	RUL,LLL: AC, metastasis	6	R: 4, L: 5	None
4	RLL: wedge, LLL: segmentectomy	RLL,LLL: AC, metastasis	6	R: 3, L: 5	None
5	RUL, RLL, LLL: wedge	RUL,RLL, LLL: HCC, metastasis	4	R: 2, L: 3	None
6	RML: wedge, LUL: segmentectomy	RML: inflammation, LUL: HCC,metastasis	4	R: 1, L: 2	None
7	RLL: wedge, LUL: wedge	RLL,LUL: AC, metastasis	5	R: 2, L: 4	None

*LoS* length of hospital stay, *RUL* right upper lobe, *RML* right middle lobe, *RLL* right lower lobe, *LUL* left upper lobe, *LLL* left lower lobe, *SqCC* Squamous cell carcinoma, *AIS* adenocarcinoma in situ, *AC* adenocarcinoma, *HCC* hepatocellular carcinoma

No surgery-related mortality was observed.

## Discussion

We introduced a single center experience of 7 successful bilateral iVATS procedures on bilateral lung nodules. This study presented some potential benefits of bilateral iVATS surgery: (1) successful and complete resections of bilateral lung nodules in a single anesthesia procedure in one hybrid operating room, (2) dramatic decreases in operation time and surgical risk (e.g., hemothorax or pneumothorax related to inadequate puncture) due to no need to transport patients from the CT procedure room to the operating room, especially for patients with bilateral lung lesions, and (3) generally optimal postoperative outcomes (e.g., acceptable length of hospital stay and chest tube duration). Furthermore, one-time bilateral localization with traditional CT guiding techniques is not feasible because possible catastrophic bilateral pneumothorax or hemothorax might occur after the localization procedure. Even if these complications did not happen, in the traditional way patients have to be hospitalized twice and receive surgery twice for their bilateral lung lesions. All these problems can be minimized or waived by applying our novel bilateral iVATS approach.

Several studies had described image-guided video assisted thoracoscopic surgery with marking in real time, in single suite, and immediate resection in a hybrid operating room. Those trials summarized many advantages of iVATS, such as (1) iVATS provides a less invasive approach and results in less discomfort for patients and improved postoperative recovery and general satisfaction; (2) complete and minimal resection of early lung cancer with optimal margin, allowing maximal preservation of patients’ lung volume and function; (3) single use of anesthesia and the use of only one room improve both perioperative outcomes (e.g., shorter operative duration and length of hospital stay) and general cost-effectiveness; and (4) iVATS encourages early cancer treatment [1–6]. However, none of these studies demonstrated the utility of iVATS for bilateral lung nodule resection. We believed this is the first case series describing bilateral iVATS with optimal clinical outcomes.

The marker for nodule localization in this study is methylene blue because it is clearly visualized on the surface of lung parenchyma and requires no extra equipment. According to previous studies, other contrast agents with larger molecular weight and larger particle size, including indocyanine green, indigo carmine, and lipiodol, may migrate slower into tissues. The higher-density contrast agents, like iopamidol and lipiodol, have been associated with allergic reactions to the contrast medium, local inflammation at the site of injection, and contrast embolization [7, 11, 12].

All lesions in our study were partially solid or mixed ground glass, and their average size was 8.93 mm. Those features often cause difficulty in making a definite diagnosis from image-guided biopsies, and surgery may also be difficult due to it being hard to palpate lesions during surgery. With the application of iVATS procedures, especially bilateral approaches, we can expect a significant increase in the number of complete resections of early lung cancers due to advantages in time and cost effectiveness, and thus other necessary treatment would be promoted, which may lead to improved overall cancer-related mortality.

Some limitations of our study are considered. First, the relatively small sample size may result in inevitable bias. Second, the postoperative follow-up periods were limited to being within the hospitalization period. Furthermore, a longer follow-up duration for a larger study group should be used to further explore the clinical outcomes of cancer-related morbidity and mortality. Finally, detailed cost-effectiveness analyses comparing bilateral iVATS with other bilateral techniques or iVATS of unilateral lung nodules should also be performed.

## Conclusion

For bilateral pulmonary nodules, the iVATS procedure seems to be a feasible and cost-effective approach.

## Data Availability

The datasets used and analysed during the current study are available from the corresponding author on reasonable request.

## Abbreviations

iVATS: image-guided video-assisted thoracoscopic surgery
CT: computed tomography
GGOs: ground glass opacities

## References

[1] Gill RR, Zheng Y, Barlow JS, Jayender J, Girard EE, Hartigan PM, Chirieac LR, Belle-King CJ, Murray K, Sears C, Wee JO, Jaklitsch MT, Colson YL, Bueno R (2015). Image-guided Video Assisted Thoracoscopic Surgery (iVATS) - Phase I-II Clinical Trial. J Surg Oncol.

[2] Yang SM, Ko WC, Lin MW, Hsu HH, Chan CY, Wu IH, Chang YC, Chen JS (2016). Image-guided thoracoscopic surgery with dye localization in a hybrid operating room. J Thorac Dis.

[3] Chen PH, Hsu HH, Yang SM, Tsai TM, Tsou KC, Liao HC, Lin MW, Chen JS (2018). Preoperative Dye Localization for Thoracoscopic Lung Surgery: Hybrid Versus Computed Tomography Room. Ann Thorac Surg.

[4] Chao YK, Wen CT, Fang HY, Hsieh MJ (2018). A single-center experience of 100 image-guided video-assisted thoracoscopic surgery procedures. J Thorac Dis.

[5] Hsieh MJ, Wen CT, Fang HY, Wen YW, Lin CC, Chao YK (2018). Learning curve of image-guided video-assisted thoracoscopic surgery for small pulmonary nodules: A prospective analysis of 30 initial patients. J Thorac Cardiovasc Surg.

[6] Chao YK, Leow OQY, Wen CT, Fang HY (2019). Image-guided thoracoscopic lung resection using a dual-marker localization technique in a hybrid operating room. Surg Endosc.

[7] Thistlethwaite PA, Gower JR, Hernandez M, Zhang Y, Picel AC, Roberts AC (2018). Needle localization of small pulmonary nodules: Lessons learned. J Thorac Cardiovasc Surg.

[8] Ujiie H, Kato T, Hu HP, Patel P, Wada H, Fujino K, Weersink R, Nguyen E, Cypel M, Pierre A, de Perrot M, Darling G, Waddell TK, Keshavjee S, Yasufuku K (2017). A novel minimally invasive near-infrared thoracoscopic localization technique of small pulmonary nodules: A phase I feasibility trial. J Thorac Cardiovasc Surg.

[9] Suzuki K, Shimohira M, Hashizume T, Ozawa Y, Sobue R, Mimura M, Mori Y, Ijima H, Watanabe K, Yano M, Yoshioka H, Shibamoto Y (2014). Usefulness of CT-guided hookwire marking before video-assisted thoracoscopic surgery for small pulmonary lesions. J Med Imaging Radiat Oncol.

[10] Bertolaccini L, Terzi A, Spada E, Acchiardi F, Ghirardo D (2012). Not palpable? Role of radio-guided video-assisted thoracic surgery for nonpalpable solitary pulmonary nodules. Gen Thorac Cardiovasc Surg.

[11] Watanabe K, Nomori H, Ohtsuka T, Kaji M, Naruke T, Suemasu K (2006). Usefulness and complications of computed tomography-guided lipiodol marking for fluoroscopy-assisted thoracoscopic resection of small pulmonary nodules: experience with 174 nodules. J Thorac Cardiovasc Surg.

[12] Nomori H, Horio H, Naruke T, Suemasu K (2002). Fluoroscopy-assisted thoracoscopic resection of lung nodules marked with lipiodol. Ann Thorac Surg.

